# Wound outcome in combat injuries is associated with a unique set of protein biomarkers

**DOI:** 10.1186/1479-5876-11-281

**Published:** 2013-11-06

**Authors:** Brett A Chromy, Angela Eldridge, Jonathan A Forsberg, Trevor S Brown, Benjamin C Kirkup, Crystal Jaing, Nicholas A Be, Eric Elster, Paul A Luciw

**Affiliations:** 1 Department of Pathology and Laboratory Medicine, School of Medicine, University of California at Davis, Davis, CA, USA; 2 Center for Comparative Medicine, University of California at Davis, Davis, CA, USA; 3 Department of Orthopaedics, National Military Medical Center, Bethesda, MD, USA; 4 Regenerative Medicine Department, Naval Medical Research Center, Silver Spring, MD, USA; 5 Department of Wound Infections, Walter Reed Army Institute of Research, Silver Spring, MD, USA; 6 Department of Medicine, F Edward Hebert School of Medicine, Uniformed Services University of the Health Sciences, Bethesda, MD, USA; 7 Physical & Life Sciences Directorate, Lawrence Livermore National Laboratory, Livermore, CA, USA; 8 Norman M. Rich Department of Surgery, Uniformed Services University of the Health Sciences, Bethesda, MD, USA

**Keywords:** Traumatic wound, Proteomics, 2-D DIGE, Wound effluent, Biomarker discovery, Wound dehiscence

## Abstract

**Background:**

The ability to forecast whether a wound will heal after closure without further debridement(s), would provide substantial benefits to patients with severe extremity trauma.

**Methods:**

Wound effluent is a readily available material which can be collected without disturbing healthy tissue. For analysis of potential host response biomarkers, forty four serial combat wound effluent samples from 19 patients with either healing or failing traumatic- and other combat-related wounds were examined by 2-D DIGE. Spot map patterns were correlated to eventual wound outcome (healed or wound failure) and analyzed using DeCyder 7.0 and differential proteins identified via LC-MS/MS.

**Results:**

This approach identified 52 protein spots that were differentially expressed and thus represent candidate biomarkers for this clinical application. Many of these proteins are intimately involved in inflammatory and immune responses. Furthermore, discriminate analysis further refined the 52 differential protein spots to a smaller subset of which successfully differentiate between wounds that will heal and those that will fail and require further surgical intervention with greater than 83% accuracy.

**Conclusion:**

These results suggest candidates for a panel of protein biomarkers that may aid traumatic wound care prognosis and treatment. We recommend that this strategy be refined, and then externally validated, in future studies of traumatic wounds.

## Introduction

The combination of effective body armor and the use of improvised explosion devices in modern warfare have greatly increased the incidence of severe survivable traumatic injuries [1-4]. Comprising approximately half of all current combat wounds, this mechanism of injury predominately affects the extremities and devastates skin, musculoskeletal tissue, the neurovascular system, and bone [4-7]. Several factors can contribute to the ability of a wound to heal or to dehisce, including the extent of injury, time from wound occurrence to care, individual immune and inflammatory responses, and microbial colonization. However, no widely used prognostic assay quantitatively predicts whether a traumatic wound will heal normally or dehisce after surgical closure.

Failure of the healing process of chronic wounds incurs massive health care costs, totaling up to $3 billion per year [8]. In general, the healing of wounds is a tightly regulated process involving hemostasis, inflammation, cell proliferation, and tissue remodeling. Wounds with impaired healing do not progress through these normal stages due to many factors including local wound characteristics, such as, oxygenation, microbial presence, and venous supply [9]. The inflammation phase is critical for the removal of contaminating organisms and, if removal is not sufficient, the remaining bacteria and endotoxins can lead to a prolonged inflammatory response [10]. One of the most commonly cited wound outcome indicators is the extent of microbial presence [5], which is clinically binned into one of three categories: contamination (non-replicating organisms present), colonization (replicating organisms present) and local infection/critical colonization (intermediate stage with local tissue response) [10]. Although microbial presence is associated with wound outcome, current clinical microbiology is not sufficient to predict non-healing, therefore, alternative methods need to be explored.

Current research on wound outcome focuses on correlating changes in levels of certain host proteins, including chemokines, cytokines, metalloproteinases and other proteases, and inflammatory marker proteins; these studies indicate that poor combat wound healing is associated with dysregulation of the inflammatory response [5,11-13]. In this report, we expand upon previous effort by describing a comprehensive study that aims to analyze the wound effluent proteome and provide a preliminary biomarker panel of proteins that can differentiate between uneventful healing and wound failure (such as dehiscence, failure of graft or flap, removal of biologic matrix) after surgical closure. If successful, these discriminating proteins can be further developed for use as a prognostic tool to aid physicians in predicting wound outcome and thus providing optimal care for traumatic- and otherwise combat-related injuries.

## Materials and methods

### Patients and sample collection

The study methodology is as reported elsewhere [5,11] and is reiterated here for completeness. In brief, serial samples were collected in an observational study with prospective data collection in accordance with the institutional review board of the Walter Reed National Military Medical Center (Bethesda, MD). All service members evacuated to the National Capital Area from Iraq and Afghanistan that had sustained high-energy penetrating injuries to one or more extremities and were without confounding co-morbid conditions, such as immune disorders, connective tissue disorders, or any conditions requiring immunosuppressive agents, were eligible for inclusion. Surgical debridement, lavage, and negative-pressure wound therapy (NPWT) were repeated every 48–72 hours until surgical wound closure or coverage at the discretion of the attending surgeon and in accordance with current institutional standards of practice. Patients were followed throughout their recovery for a minimum of six weeks to determine whether their wounds healed normally or dehisced (Table 1).

**Table 1:** Patient demographics and clinical characteristics

**Patient Demographics and Clinical Characteristics**	**Healed**	**Dehisced**
	***n *** **= 21**	***n *** **= 23**
**Age, y**	23.7 ± 3.8	22 ± 3.2
**BMI, mean (SD)**	26 ± 2.4	25 ± 3.8
**Gender, *n *** **(%)**		
Male	21 (100%)	23 (100%)
Female	0 (0%)	0 (0%)
**Wound Location, *n***		
Upper Body	19	0
Lower Body	2	23
**Wound Type, *n***		
Soft Tissue Infection	12	17
Fasciotomy	5	7
Amputation	3	0
Open Fracture	1	0
**Type of Closure, *n***		
Primary	13	9
Integra	2	12
Graft	4	1
Flap	2	1
**Injury Severity Score, mean (SD)**	20 ± 8.3	17 ± 7.3
**No. of Total Surgeries, mean (SD)**	3.8 ± 1.2	3.6 ± 1.4
**Presence of Traumatic Brain Injury, *n***		
Yes	16	7
No	2	13
Unknown	3	3
**Presence of Heterotrophic Ossification, *n***		
Yes	16	21
No	5	2
**Days from Injury to Arrival at Facility, mean (SD)**	4.2 ± 1.6	4.4 ± 1.4
**Days from Injury to Wound Closure, mean (SD)**	12.1 ± 3.8	11.7 ± 4.9
**Days from Injury to Sample Collection, mean (SD)**	6.2 ± 1.4	6.6 ± 1.5
**Days from Sample Collection to Closure, mean (SD)**	5.9 ± 3.8	5.0 ± 4.5

The demographic and clinical characteristics of the 20 patients that comprised the 44 wound effluent samples are displayed.

Wound effluent samples (≥30 ml) were collected from the NPWT canister (without gel pack; Kinetic Concepts, Inc., San Antonio, TX) over a 12-hour period prior to each wound debridement and 2 hours following the first surgical debridement and subsequent surgical debridements. The samples used in this study were collected from up to three wounds for up to three serial debridements. Samples were treated like serum and centrifuged at 2500 x g for 10 minutes to remove particulate matter and emboli. Effluent supernatants were transferred to individually labeled polypropylene tubes, flash-frozen in liquid nitrogen, and stored at -80°C until analysis.

### Removal of highly abundant proteins

Depletion of high abundant proteins was performed according to manufacturer’s instructions. Briefly, patient wound effluent was diluted five fold in Buffer A (Agilent Technologies) into 200 μl total volume and centrifuged through a 0.22 micron spin filter (Millipore) tube at 16,000 x g for 5 min to remove particulates. Then effluent fluids were processed using Multiple Affinity Removal Column Human-6 (Agilent Technologies), which specifically removes albumin, IgA, IgG, antitrypsin, transferrin and haptoglobin. A low abundant protein fraction was collected for each sample. Fractions were concentrated by precipitation using an equal volume of 20% trichloroacetic acid solution and incubated at 4°C for 30 min. The precipitate was washed twice with cold 100% acetone, allowed to air dry, and then resuspended in DIGE labeling buffer (7 M urea, 2 M thiourea, 4% CHAPS, 30 mM Tris, pH 8.5). Protein quantification was performed using Precision Red Advanced Protein Assay Reagent (Cytoskeleton Inc.).

### 2-D DIGE analysis

Crude and high abundant protein depleted effluent samples were separated in 2 dimensions according to GE Life Sciences Ettan DIGE system protocol. Briefly, each sample (50 μg) was minimally labeled with 200 pmol Cy3 or Cy5 for 30 min, a pooled standard of all 44 experimental samples were labeled similarly with Cy2. All labeling reactions were stopped by the addition of 1 μl of 1 mM lysine. Individually labeled samples were pooled and added to rehydration buffer (7 M urea, 2 M thiourea, 4% CHAPS, 1.2% destreak, 1% pharmalytes). A final volume of 450 μl sample was loaded onto 24 cm pH3-10NL Immobiline DryStrips (GE Life Sciences) and focused by active overnight rehydration, followed by active isoelectric focusing for a total of 62,500 Vhrs. Strips were equilibrated in SDS equilibration buffer (6 M urea, 30% glycerol, 2% SDS) for 15 min with 10 mg/ml DTT, then 15 min in fresh buffer with 25 mg/ml 15 min with iodoacetamide, then applied to DIGE gels (GE Life Sciences) for 2nd dimension separation. The resulting CyDye labeled protein gels were scanned using 100 micron resolution on Typhoon 9410 (GE Life Sciences).

### DeCyder multivariate analysis

Data analysis was carried out using the various modules of DeCyder 2-D 7.0 software (GE Life Sciences). The difference in-gel analysis (DIA) module was used to determine the optimal and average spot detection settings. All 22 gel images were given to the batch processor module with the designated settings to generate spot maps using 2,500 as the estimated number of spots. Cy2 labeled pooled standard was used to normalize spot intensity within each gel. The spot map with the greatest number of detected spots was set as the master gel, and biological variation analysis (BVA) module was then used for automated spot matching across all the gels. Gel matching quality was manually verified, and landmarks were added where improved matching quality was needed. The extended data analysis (EDA) software package (GE Life Sciences) was used for differential protein determination, PCA, hierarchical clustering, k-means partitioning analysis and discriminate analysis calculations [14].

For identification of differential proteins, each sample spot map was assembled into the appropriate experimental group (healed or dehisced wounds), and the average ratio fold-change were calculated. A base set was established using only spots that were matched on greater than 60% of the spot maps. Spots having fold change > 1.2 and p-value < 0.05 (according to [15]) were considered differentially expressed, each spot was manually verified for an acceptable three dimensional characteristic protein profile and for adequate material for subsequent mass spectrometry identification. Spots not meeting these criteria were excluded from further analysis. The set of confirmed differential proteins for each of the experimental group comparisons was used for PCA, hierarchical clustering (heat map and k-means calculations) and discriminate analysis.

### Protein digestion and identification

Differential proteins were excised from a preparative gel with additional protein for identification purposes. Excised gel pieces were destained in 100 mM ammonium bicarbonate for 1 h at room temperature, dehydrated with successive 100% acetonitrile washes and dried in a SpeedVac for 30 min. The gel pieces were then rehydrated with 130 ng modified porcine trypsin (Promega) in 50 mM ammonium bicarbonate and incubated for 16 h at 37°C. Supernatants were collected and peptides further extracted with 5% trifluoroacetic acid in 50% acetonitrile, supernatants and extraction fluid were pooled together. Tryptic peptides were concentrated down to 5 μl by SpeedVac and analyzed by a LC-MS/MS LTQ-Orbitrap using nanoflow HPLC with a HALO C18 reversed phase separation column (Bruker-Michrom). The resulting peak lists were searched against the Human International Protein Index database using the MASCOT search engine according to the following parameters: up to two missed cleavages, peptide mass tolerance of 1.2 Da, fragment mass tolerance of 0.6 Da, fixed modification carbamidation, and variable modification oxidation (M). Protein identifications with probability score of 95% or higher and contain at least two unique peptides were considered valid.

### Functional classification

A list of UniProt IDs for the 45 identified differential spots among healed versus dehisced wounds were submitted to the GORetriever online tool to retrieve their GO annotations. Corresponding GO annotations and online plugin tool CateGOrizer were used to categorize the proteins according to Immune System Gene class classification list, producing a pie chart to display the distribution of involved functions.

## Results

In this study, we characterized the proteome of traumatic wound patient effluent samples (n = 44). Effluent was collected at the Naval Medical Research Center using NPWT, which uses a vacuum system to enable earlier wound closure.

All effluent samples were subjected to removal of high abundant proteins to improve overall spot clarity and separated by 2-D DIGE according to protocols established by Chromy et al. [16]. Spot maps were analyzed by DeCyder 7.0 by which a total of 1800 spots were detected and quantified in each the 22 gels using the DIA module, and gel-to-gel spot matching was performed using the BVA module. This approach yielded a significant increase in the number of discernible protein spots when compared to a similar analysis of crude wound effluent, which lead to increased sensitivity and opportunity to find novel protein biomarkers.

### Differential protein spot determination

**Figure 1:**
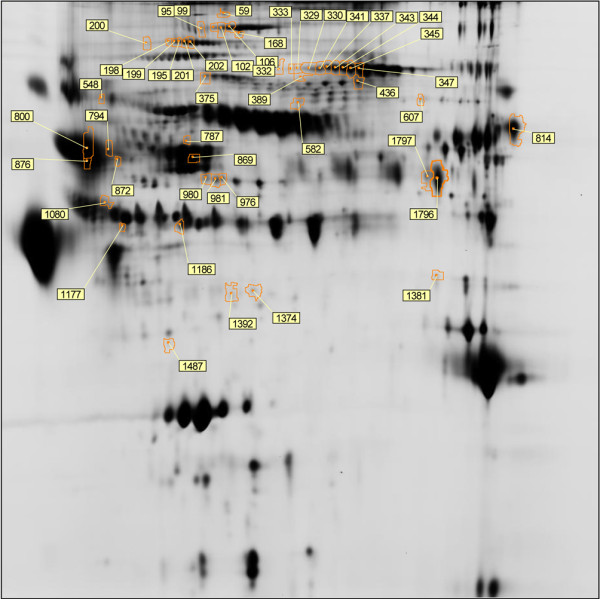
**Differential expression analysis.** A total of 52 unique protein spots with greater than 1.2 fold change and p-value < 0.05. Each spot boundary is defined in orange and labeled with assigned spot number. Differential spots were excised from the gel and identified *via* mass spectrometry.

Differential protein spots were matched on greater than 60% of the spot maps, had a fold change > 1.2 [15] with a p-value < 0.05. A total of 52 unique protein spots were determined to be differential; their distribution on the preparative pick gel is shown in Figure 1. The 52 differential spots were excised and 45 spots were confidently identified yielding 25 unique proteins. Assigned spot number, IPI database number, gene/protein name, MASCOT score, fold change/t-test along with theoretical molecular weight and pI are displayed for each identified protein in Table 2. Many areas on the gel show several spots in a horizontal line and were determined to be the same protein with slight variations in their pI [17]. Because only some of the spots in the line were determined to be differential, these data indicate that post-translational modifications of specific proteins are important in discriminating between healed and dehisced effluent samples.

**Table 2:** Proteins showing differential responses

**Spot No.**	**IPI No.**	**Gene Name**	**Protein Name**	**Mascot Score**	**Fold Change (Healed/ Dehisced)**	**T-test**	**Th. MW**	**Th. pI**
59	IPI00440484	PER1	Period Circadian protein homolog 1	133	-1.23	0.0414	137667	5.73
95*	IPI00645206	PCDH17	Isoform 1 of Protocadherin 17	131	-1.22	0.0121	127405	5.03
99*	IPI00235407	STK36	Isoform 1 of Serine/threonine protein kinase 3	142	-1.24	0.0018	145728	5.57
102	IPI00185661	USP32	Ubiquitin carboxyl terminal hydrolase 32	125	-1.2	0.0164	145671	6.18
106	IPI00470744	PARG	Isoform 2 of Poly (ADP-ribose) glycohydrolase	113	-1.22	0.0106	103503	5.58
168*	IPI00290328	PTPRJ	Receptor type tyrosine protein phosphotase precursor	130	-1.51	0.0061	152960	5.8
195*	IPI00017601	CP	Ceruloplasmin	101	-1.27	0.0116	122983	5.44
198*	IPI00017601	CP	Ceruloplasmin	121	-1.39	0.0076	122983	5.44
199	IPI00017601	CP	Ceruloplasmin	182	-1.31	0.0095	122983	5.44
200	IPI00017601	CP	Ceruloplasmin	116	-1.33	0.0075	122983	5.44
201	IPI00017601	CP	Ceruloplasmin	112	-1.3	0.0254	122983	5.44
202	IPI00017601	CP	Ceruloplasmin	154	-1.31	0.0130	122983	5.44
329	IPI00011736	PIK3R2	Phosphatidylinositol 3 kinase regulatory subunit 2	88	1.26	0.0259	81837	6.03
330	IPI00783987	C3	Complement C3	115	1.55	0.0003	188569	6.02
333	IPI00011736	PIK3R2	Phosphatidylinositol 3 kinase regulatory subunit 2	111	1.25	0.0046	81837	6.03
337	IPI00783987	C3	Complement C3	88	1.53	0.0104	188569	6.02
341*	IPI00783987	C3	Complement C3	147	1.56	0.0006	188569	6.02
343*	IPI00783987	C3	Complement C3	189	1.62	0.0006	188569	6.02
344	IPI00783987	C3	Complement C3	120	1.69	0.0048	188569	6.02
345	IPI00783987	C3	Complement C3	79	1.59	0.0125	188569	6.02
347	IPI00783987	C3	Complement C3	104	1.52	0.0339	188569	6.02
375*	IPI00607814	XPNPEP1	Xaa-Pro aminopeptidase 1 Isoform 2	121	1.23	0.0415	72746	5.67
389	IPI00157535	EPS8L1	Isoform 4 of Epigrowth factor receptor kinase subunit 8	118	1.23	0.0168	88920	6.49
436	IPI00009089	FRS3	Fibroblast growth factor receptor substrate 3	121	-1.28	0.0198	55169	6.81
582	IPI00301255	IGSF21	Immunoglobulin superfamily member 21	109	-1.23	0.0480	51855	6.48
602	IPI00329555	F7	Isoform A of Coagulation factor VII	178	-1.54	0.0232	53043	6.91
607	IPI00329555	F7	Isoform A of Coagulation factor VII	130	-1.37	0.0462	53043	6.91
787	IPI00021891	FGG	Fibrinogen gamma chain 1	135	1.33	0.0267	51511	5.37
794	IPI00847635	SERPINA3	Isoform 1 of alpha-1-antichymotrypsin	146	1.57	0.0067	47792	5.33
800	IPI00847635	SERPINA3	Isoform 1 of alpha-1-antichymotrypsin	216	1.22	0.0273	47792	5.33
814	IPI00219330	ILF3	Isoform 5 of Interleukin enhancer binding factor 3	252	-1.35	0.0205	74959	8.4
869	IPI00783625	SERPINB5	Isoform 1 of Serpin B5	120	1.27	0.0356	42530	5.72
872*	IPI00847635	SERPINA3	Isoform 1 of alpha-1-antichymotrypsin	111	1.39	0.0456	47792	5.33
876	IPI00847635	SERPINA3	Isoform 1 of alpha-1-antichymotrypsin	283	1.24	0.0424	47792	5.33
976	IPI00004657	HLA-B	HLA class 1 histocompatibility antigen	102	1.27	0.0150	40777	5.57
980	IPI00004657	HLA-B	HLA class 1 histocompatibility antigen	121	1.34	0.0383	40777	5.57
981	IPI00004657	HLA-B	HLA class 1 histocompatibility antigen	101	1.57	0.0008	40777	5.57
1080	IPI00166729	AZGP1	Zinc alpha 2 glycoprotein precursor	147	-1.34	0.0031	34258	5.71
1177	IPI00555812	GC	Vitamin D binding protein precursor	104	-1.28	0.0151	52963	5.4
1186	IPI00641737	HP	Haptoglobin	92	-1.29	0.0374	45205	6.13
1374	IPI00978715	CLU	Clusterin	109	-1.25	0.0106	52495	5.88
1381	IPI00410313	KIR3KL3	Killer cell immunoglobulin like receptor 3DL3	132	1.25	0.0144	45470	7.27
1392	IPI00022391	APCS	Serum Amyloid P component	181	1.48	0.0223	25837	6.1
1487	IPI00386246	AMPH	Amphiphysin I variant CT3, fragment	119	1.24	0.0251	31195	4.23
1796	IPI00007879	SRF	Serum response factor	132	1.21	0.0460	51592	7.83

The 45 identified differential spots found among biological replicates of healed versus dehisced wounds (corresponding to t-test < 0.05 and fold change cutoff > 1.2) were identified via LC-MS/MS and yieled 25 unique protein identifications. The table includes spot number, IPI database number, gene name, protein name, MASCOT identification score, fold change and t-test of expression between healed and dehisced wounds, theoretical molecular weight and pI. *Indicates the 9 proteins selected as markers by discriminate analysis. Spot numbers 332, 336, 548, 763, 978, and 1797 were unable to be confidently identified.

### PCA and hierarchical clustering

**Figure 2:**
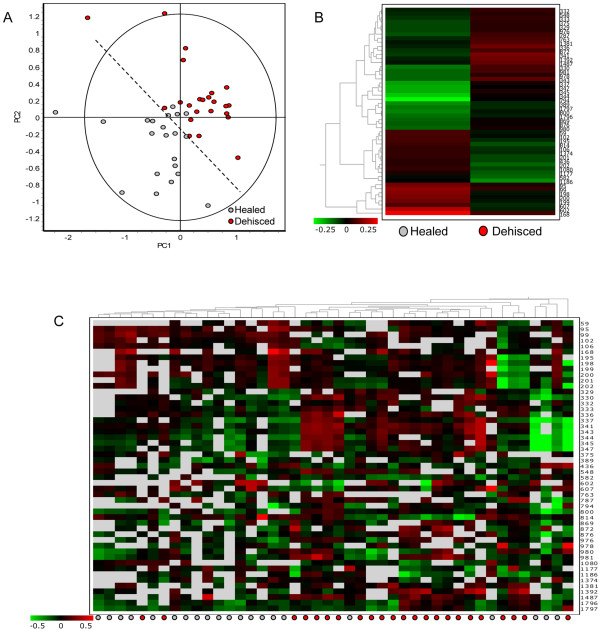
**DeCyder cluster analysis.** Fifty two verified differential expression protein spots (t-test > 0.5, fold change > 1.2) were used for the following analyses using DeCyder Extended Data Analysis. **(A)** Principle component analysis shows good separation between healed and dehisced samples; each dot represents an individual gel spot map. **(B)** Hierarchical clustering using the average abundance of differential spots for each experimental group, experimental groups are displayed in columns and protein spots by row, differential expression is displayed by color (red being up-regulated and green being down-regulated relative to the pooled standard. Clusters of differential proteins showing similar expression patterns are further defined in Figure 4. **(C)** Hierarchical clustering of 44 spot maps according to the 52 differential protein spots, individual patient spot maps are displayed by column and protein spots by rows.

The 52 verified differential proteins were used for PCA of the wound effluent sample spot maps. Figure 2A shows good separation between healed and dehisced samples, supporting the theory that the host proteome can be used to differentiate and possibly predict wound outcome. Figure 2B shows hierarchical clustering of the differential proteins, using the average abundance of each spot within each experimental group. Figure 2C was produced by cluster analysis using the 52 differential spots; the spot maps showing similar protein expression patterns are clustered together. Similar to the PCA result, hierarchical clustering adequately separated healed spot maps from dehisced spot maps.

### K-means protein clusters and functions

**Figure 3:**
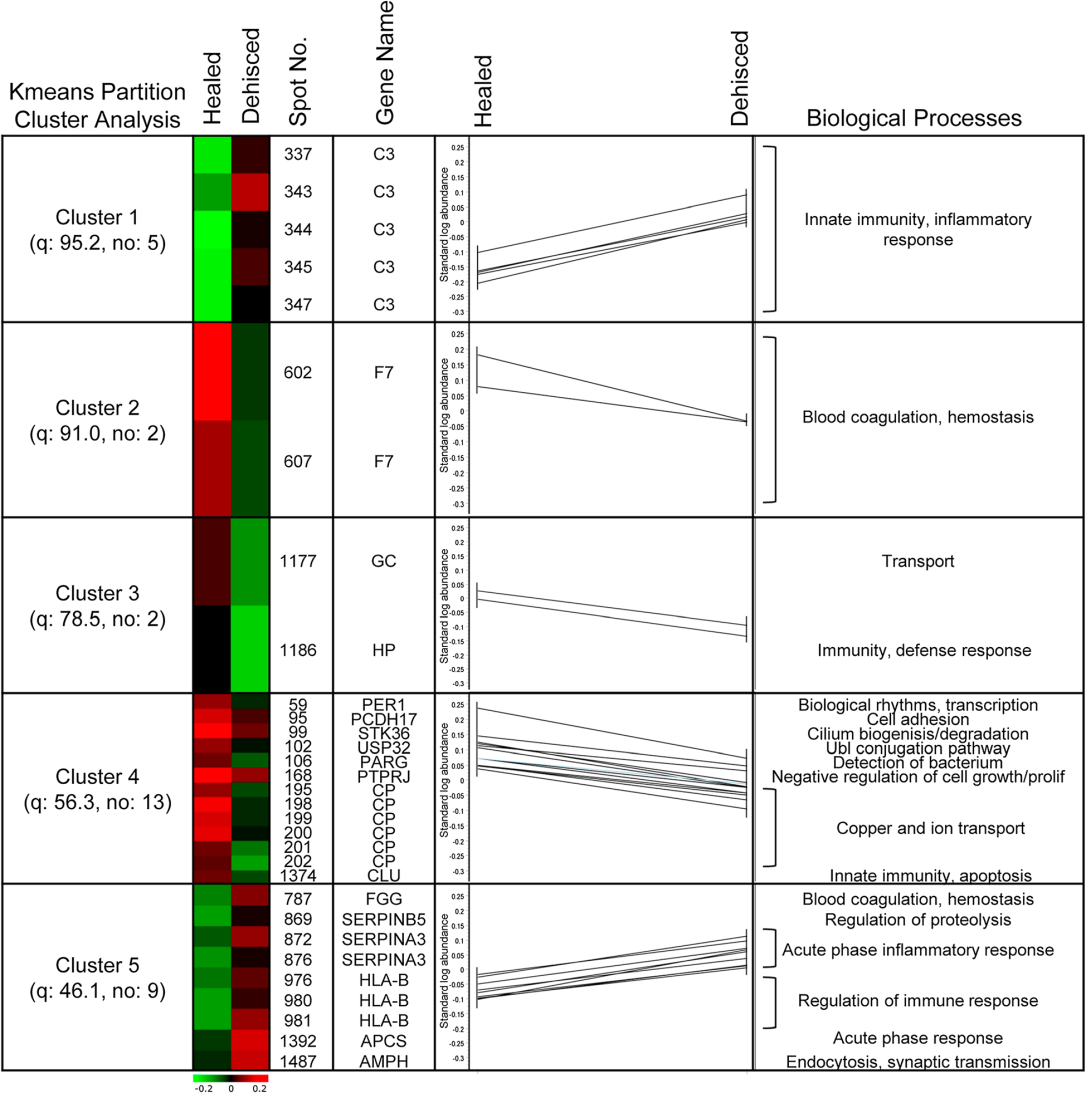
**K-means partitioning analysis.** Five clusters of proteins spots with similar expression patterns. Spot abundance, identification and known functional category are displayed to expose biological processes involved in distinguishing between healed and dehiscing wounds.

K-means partitioning analysis, within the DeCyder EDA module, exposes protein spot clusters exhibiting similar expression patterns (Figure 3). Interestingly, many of these proteins regulate different aspects of immunity and inflammatory responses. Specifically, cluster 1 is comprised of 5 (spot no. 337, 343, 344, 345, 347) of 12 separate spots with nearly identical molecular weights and slightly shifted pI values, encompassing the left half of the horizontal spot streak. All spots were identified as complement C3 protein and are individually, as well as collectively, upregulated in dehisced wound effluent samples. This acidic pI shift is commonly caused by post translational modifications (PTMs) [18], such as phosphorylation, which is a key regulatory mechanism in most systemic responses [19] that alters activation state of proteins [17]. In particular, phosphorylation has been shown to activate complement C3 by increasing complement binding [20] and opsonization [21] of invading pathogens, and is involved in anti-inflammatory regulatory mechanisms [22]. These data suggest that not only the overall protein expression level is important, but the abundance of certain isoforms that are a result of specific PTMs can help discriminate between healed and dehisced wound effluent.

**Figure 4:**
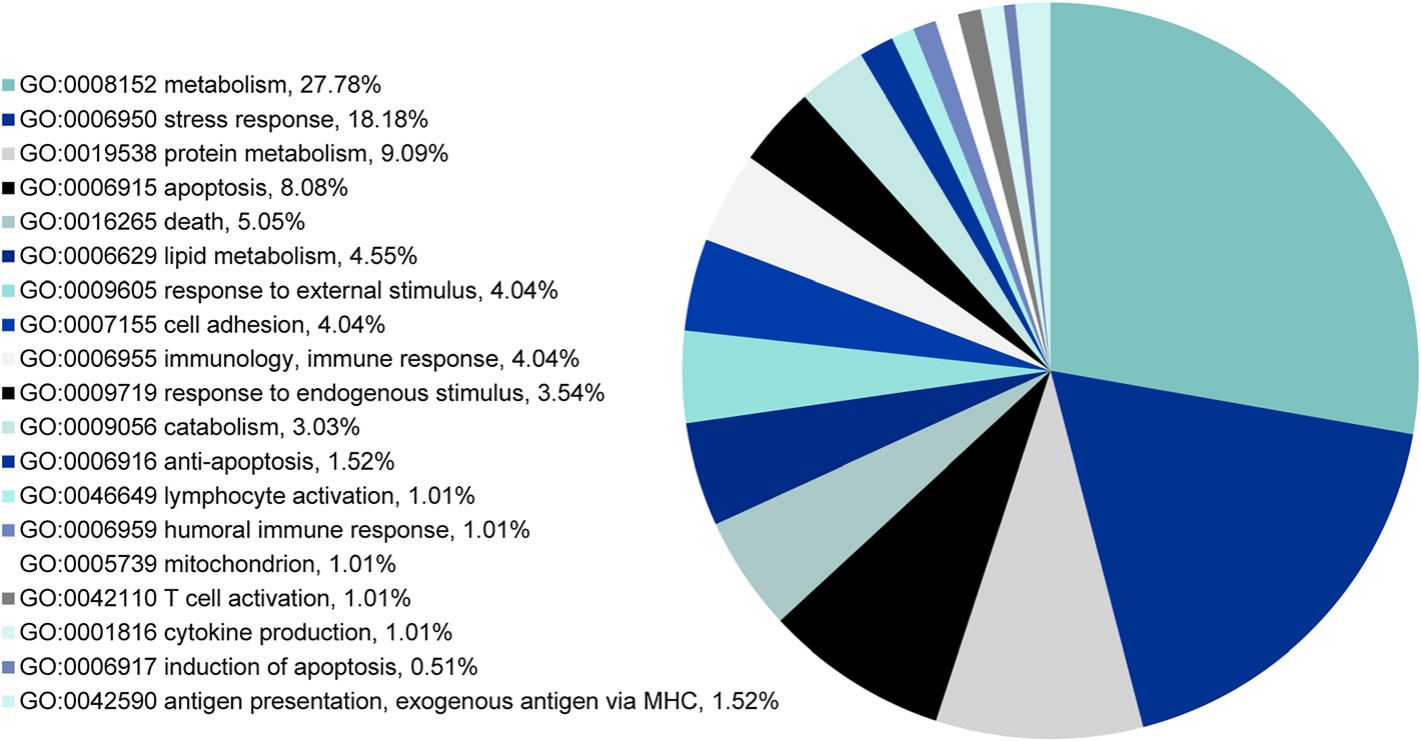
**Functional classifications.** The list of 45 identified differential proteins were functionally categorized into classes according to the Immune System Gene classification list using the CateGOrizer online tool.

The set of 25 unique differential proteins were functionally categorized using CateGOrizer according to the Immune System gene classes classification list (Figure 4). The largest functional class is metabolism, which is consistent with a large number of proteins having some involvement in cellular metabolism. The second largest category is stress response, which relates to the systemic impact of a major wound on the patient. These data strongly suggest that host proteins involved in responding to stress are changing abundance according to wound outcome. Moreover, these results are consistent with previous studies showing the importance of the stress response in wound healing [23-26].

### Discriminate analysis

Patterns of differentially expressed proteins were analyzed *via* the EDA module to determine the appropriate biomarker panel that effectively discriminates between healed and dehisced wound effluent (Table 3). Panel A displays the 9 selected protein biomarkers with the respective discrimination accuracy (83.83% ± 2.8) and Panel B lists their gene names and protein identifications. These markers include protocadherin 17, serine/threonine protein kinase 3, receptor type tyrosine protein phosphotase precursor, ceruloplasmin (2 spots), complement C3 (2 spots), xaa-pro aminopeptidase 1, and alpha-1-antichymotrypsin. Most importantly, these proteins could be used in a biomarker panel to determine which sample a particular host proteome belongs to and be further developed for use in a prognostic protein panel to aid physicians in selecting appropriate traumatic wound trauma treatment. Thus, 2-D DIGE data have revealed a set of protein biomarkers which can effectively discriminate between healed and dehisced wound effluent samples.

**Table 3:** Discriminate analysis

**A. Comparison (left vs. right)**		**Differential Proteins**	**Markers selected**	**Accuracy (%)**
Healed	Dehisced	52	9	83.83 ± 2.8
**B. Healed vs. Dehisced Discriminate Markers**				
**Spot No.**	**Gene Name**	**Protein Name**		
95	PCH17	Protocadherin 17		
99	STK36	Serine/threonine protein kinase 3		
168	PTPRJ	Receptor type tyrosine protein phosphotase precursor		
195	CP	Ceruloplasmin		
198	CP	Ceruloplasmin		
341	C3	Complement C3		
343	C3	Complement C3		
375	XPNPEP1	Xaa-Pro aminopeptidase 1		
872	SERPINA3	Alpha-1-antichymotrypsin		

Differential proteins were used to determine the appropriate marker selection panel of proteins that effectively discriminate between healed and dehisced effluent samples. A total of 9 markers were selected which give greater than 83% accuracy (panel A), the corresponding protein identifications are shown in panel B.

## Discussion

Initial treatment of traumatic- and other combat-related injuries involves aggressive resuscitation measures in conjunction with debridement procedures that are geared towards achieving hemostasis and the removal of devitalized tissue. Wounds are treated with negative pressure wound therapy, and left open for subsequent serial debridement procedures. This is necessary because the zone of injury in traumatic- and other high-energy wounds may not be obvious at the outset, and evolves over time. As such, timing of definitive closure is largely subjective, as the surgeon weighs the benefits of successful wound closure, with the risk of wound failure which will necessitate further, perhaps more aggressive, debridement procedures, and can ultimately jeopardize fracture fixation and/or current levels of amputation. In addition, treatment of failed wounds prolongs the hospital length of stay and delays rehabilitation, resulting in an increase of healthcare costs to both the payer and patient. The main cause for this subjective decision is the lack of techniques that can objectively assess the suitability of a wound for closure and/or accurately portend successful wound healing [27].

Wound effluent (exudate) has proven to be a rich source of protein biomarkers that are associated with wound healing outcome in other types of extremity wounds. Two research groups have used a similar gel electrophoresis-based technique, one dimensional SDS-PAGE followed by band excision and LC-MS/MS protein identification, to analyze the proteome of wound effluent. Eming et al. [28] analyzed patient effluent from healing and nonhealing venous leg ulcer wounds and identified 149 differentially expressed proteins. Many of these proteins are known to be involved in persistent inflammatory or tissue destruction responses which allow for a better understanding of disease progression in this type of wound. Escalante et al. [29] analyzed the proteome of effluent collected from mice injected with 2 different types of snake venom enzymes. Their results showed a total of 78 differentially expressed proteins (10 serum proteins, 34 cytosolic proteins, 16 keratins, 2 macroglobulins, and 16 extracellular matrix proteins) which provided novel information on the tissue damaging pathogenic mechanisms of these toxins and the subsequently triggered inflammatory reaction. Taken together, these studies provide evidence that wound effluent directly reflects the wound site microenvironment and is, therefore, a suitable candidate for identifying protein biomarkers that can discriminate between various stages of healing.

Other studies have used gel-based proteomics to examine wound effluent from chronic wounds. For example, Upton and coworkers, applying the same affinity chromatography used in this manuscript, found that removal of high abundant proteins enhanced the ability to detect lower abundant proteins in chronic wound fluid [30]. The same group produced a review article on the state of proteomics in chronic wound research and suggested affinity chromatography can also be beneficial to compensate for dynamic range problems normally associated with gel-based proteomics. A further suggestion to increase the dynamic range of 2D gels is to use improved pI separations. This review also suggested that using methods such as 2-D DIGE could compensate for the variable protein amounts and small clinical samples available for wound research [31]. Herein, we use these gel proteomic enhancements, namely, 2-D DIGE with large format gels that increase the pI separation and affinity chromatography to improve the dynamic range of the studied proteome. Other important literature showing proteomic characterization of chronic wound fluid comes from Wyffels and coworkers, who published two articles using different techniques (one using gels, the other using MS and protein arrays) [32,33]. Their earlier manuscript shows proteomic differences between the interior and periphery of wounds using 2DE. They also found differences between chronic and healed wounds, identifying S100A9 as a putative biomarker of wound healing. They report that 2DE is not optimal for the characterization of the protein profile of chronic wounds and alternate techniques, mass spectrometry and protein arrays, which they used in the more recent publication are needed. The use of multiple proteomic techniques for full characterization is optimal and taken together, the two papers from Wyfells and coworkers show a comprehensive approach to proteomic characterization with the use of the three main proteomic characterization techniques: gels, MS, and arrays. We support the use of these additional techniques for comprehensive proteomic characterization, however, an important distinction exists between the gel-based work in the 2010 Wyfells manuscript and ours. We use 2-D DIGE as compared to conventional 2DE. The 2-D DIGE approach uses an internal pooled standard that removes the major problem with gel-based proteomics, namely the gel to gel variation. In addition, the use of the 2-D DIGE system allows for lower level detection and increases the potential pool of putative biomarkers. Nonetheless, this literature from chronic wound fluid proteomics helps guide our future work, which will include additional proteomic techniques for more comprehensive characterization.

Current biomarker research from our group, involving traumatic and other high-energy combat wounds, is based on the analysis of serum, effluent and tissue biopsy specimens [11-13]. This work led to a unique understanding of the mechanisms involved in healing with regards to the timing of surgical wound closure. Many proinflammatory proteins and cytokines show increased expression in dehiscing wounds: procalcitonin [12] and IL-6 [11] (serum and effluent); IL-8 [11] (serum and tissue biopsy); MMP-2, MMP-3, MMP-7 [13], CCL3 [11] (serum only); IL-1 alpha/beta, CCL2, and GM-CSF [11] (tissue biopsy only). Similarly, other inflammatory mediator proteins display decreased expression in wounds that failed to heal: IL-13 (effluent [12] and tissue biopsy [11]); CCL5 [12], IL-2, inducible protein-10 [11] (effluent only); IL-4, IL-5 [11] (tissue biopsy only). The biomarkers found in this current study will be used along with these other ongoing cytokine-based immunoassay results to further the development of assays that can characterize wounds and lead to improved prognostic tools. We aim to use these prognostic tools to aid physicians in predicting wound outcome and provide optimal care for traumatic- and other combat-related injuries.

In this study, the classes of proteins that make up the highest percent of change include those involved with metabolism, stress, and cell death (Figure 4). These results are not surprising as wound healing requires substantial changes in cell growth, cell maintenance, and cell death to be completed successfully. Over 40% of the proteins that are differentially expressed involve metabolism, while almost 20% are involved in stress, and another 13+% involve apoptosis. The emphasis of differential expression on these protein classes suggests that specific pathways that lead to cell growth, autophagy, and cell death are vital for appropriate wound healing. Moreover, the pattern of protein expression, especially given sufficient redundancy for certain cell functions, may also help to characterize appropriate wound healing. Future characterization of protein expression changes with additional diverse datasets may lead to functional models that can characterize new wounds and help determine their treatment route leading to appropriate healing.

Dysfunction and dysregulation of inflammatory and immune responses as a contributing factor to determining wound outcome support the result of this wound effluent proteomic profiling study of combat wound effluent. Of the 52 differential proteins, complement C3 (C3), alpha-1-antichymotrypsin (SERPINA3), immunoglobulin superfamily member 21 (IGSF21), HLA class 1 histocompatability antigen (HLA-B), clusterin (CLU), haptoglobin (HP), serum amyloid P component (APCS) have roles in immune and/or inflammatory responses and were found to be differentially expressed between healed and dehisced wound effluent samples. In addition, two proteins involved in hemostasis showed differential expression, i.e. decrease of coagulation factor VII (F7) and increase of fibrinogen gamma chain (FGG) in dehisced samples.

Most importantly, the discriminate marker panel (Table 3) displays the 9 protein spots that can differentiate an effluent sample as healed or dehisced with an accuracy of greater than 83%. Accordingly, these proteins have the potential to be developed into a prognostic panel which can be used by physicians to determine a wound’s likeliness to heal normally or dehisce after surgical closure. Evaluating wound effluent from traumatic- and other combat wounds using the advanced proteomic method of 2-D DIGE is an important step to understanding the protein expression changes in the local wound environment.

## Conclusions

This research contributes to our development of a personalized clinical treatment of combat wounds which will help mitigate the risk of wound closure in this challenging clinical scenario, and potentially lead to improved healing outcomes while decreasing the number of surgical procedures, hospital length of stay and costs.

## Abbreviations

DIGE: Differential gel electrophoresis; LC-MS/MS: Liquid chromatography mass spectrometry mass spectrometry; NPWT: Negative-pressure wound therapy; BVA: Biological variation analysis; EDA: Extended data analysis; PCA: Principle component analysis.

## Competing interests

The authors declare that they have no competing interests of either financial or non-financial nature regarding the work described in the present manuscript and its publication.

## Authors’ contributions

BAC, AE, and PAL initially conceived of and designed the proteomics experiments. The overall design of the study, including planning and various aspects of interpretation of results, involved BAC, AE, JAF, TSB, BCK, CJ, NAB, EE and PAL. AE and BAC acquired the data. TSB, JAF and EE supplied crucial samples and clinical information. AE PAL and BAC generated the initial draft of the manuscript. BAC, AE, JAF, TSB, BCK, CJ, NAB, EE, and PAL provided critical revisions. All authors read and approved the final manuscript.

## Disclaimer

The views expressed in this manuscript are those of the authors and do not reflect the official policy of the Department of the Army, Department of the Navy, the Department of Defense or the United States Government.

This effort was supported (in part) by the U.S. Navy Bureau of Medicine and Surgery under the Medical Development Program and Office of Naval Research.

Some authors are military service members (or employees of the U.S. Government). This work was prepared as part of our official duties. Title 17 U.S.C. 105 provides the “Copyright protection under this title is not available for any work of the United States Government.” Title 17 U.S.C. 101 defines a U.S. Government work as a work prepared by a military service member or employee of the U.S. Government as part of that person’s official duties.

I/We certify that all individuals who qualify as authors have been listed; each has participated in the conception and design of this work, the analysis of data (when applicable), the writing of the document, and the approval of the submission of this version; that the document represents valid work; that if we used information derived from another source, we obtained all necessary approvals to use it and made appropriate acknowledgements in the document; and that each takes public responsibility for it.
